# Non-Genomic Androgen Action Regulates Proliferative/Migratory Signaling in Stromal Cells

**DOI:** 10.3389/fendo.2014.00225

**Published:** 2015-01-19

**Authors:** Marzia Di Donato, Pia Giovannelli, Gustavo Cernera, Annalisa Di Santi, Irene Marino, Antonio Bilancio, Giovanni Galasso, Ferdinando Auricchio, Antimo Migliaccio, Gabriella Castoria

**Affiliations:** ^1^Department of Biochemistry, Biophysics and General Pathology, II University of Naples, Naples, Italy

**Keywords:** androgen receptor, filamin A, migration, growth suppression, fibroblasts, fibrosarcoma

## Abstract

Prostate cancer (PCa) is the major cause of cancer-related death among the male population of Western society, and androgen-deprivation therapy (ADT) represents the first line in PCa treatment. However, although androgen receptor (AR) expression is maintained throughout the various stages of PCa, ADT frequently fails. Clinical studies have demonstrated that different androgen/AR signaling pathways operate in target tissues. AR stimulates growth and transformation of target cells, but under certain conditions slows down their proliferation. In this review, we discuss the role of AR in controlling different functions of mesenchymal and transformed mesenchymal cells. Findings here presented support the role of AR in suppressing proliferation and stimulating migration of stromal cells, with implications for current approaches to cancer therapy.

## Introduction

Prostate cells are the primary targets of androgens, which regulate development, growth, and function of prostate. In this organ, androgens act through the androgen receptor (AR), which is expressed in both epithelium and stroma, and studies with recombinant tissues indicate that prostatic development is mediated by stromal, but not epithelial AR (1). Sex steroids influence prostate cancer (PCa) initiation and/or progression (2), whereas androgen removal increases PCa cell death and inhibits cell proliferation. Hence, androgen-deprivation therapy (ADT) is the standard therapy for men with PCa. However, despite extensive efforts in the clinical development of PCa therapies, current treatments only control tumor growth initially, and fail to achieve long-term efficacy since most PCa patients eventually relapse.

The role of epithelial AR in PCa has been extensively studied in the last few decades. There are, however, many other AR target tissues throughout the body (3), though specific information on the role of AR in these cells is still minimal and few investigations have been undertaken to elucidate the role of stromal AR. It has been suggested that AR may direct stromal cells toward epithelial PCa cells upon an increase in local androgen levels, as frequently occurs in PCa (4). Stromal AR has also been shown to mediate PCa metastasis (5) and induce prostatic intraepithelial neoplasia (6). Nonetheless, data on the role of stromal AR still remain controversial.

AR paradoxically fosters or inhibits proliferation of target cells depending on cell type, microenvironment, and hormone levels (7). Reciprocal AR responses in epithelium and stroma regulate prostate development and homeostasis. Aberrant responses might result in tumorigenesis. Androgen binding to AR induces proliferation in PCa epithelial cells and differentiation in normal prostate epithelial cells (8). Prostate stromal cells express AR, but their growth is insensitive to androgen stimulation (9). Fibroblasts and human fibrosarcoma HT1080 cells do not grow in response to 10 nM androgen. At this hormone concentration, these cells undergo migration as a consequence of a bipartite AR/filamin A (FlnA) complex assembly (10, 11).

The dichotomous response (migration/proliferation) of fibroblasts to different androgen concentrations mimics that of growth factors, which trigger motility or proliferation [reviewed in Ref. (12)]. A flurry of reports has investigated the molecular basis for a cell decision to “go or grow.” EGF, VEGF, and PDGF trigger motility or proliferation depending on cell type, receptor distribution or internalization, ligand concentration, and dynamic regulation of signaling networks (13–17). In NIH3T3 cells, for instance, low PDGF concentration induces migration, whereas high concentration triggers proliferation. Cells make these different decisions as a consequence of the different endocytic routes (clathrin- and non-clathrin-mediated) engaged by the PDGF receptor (18). A G-alpha i-GIV complex binds EGF-R and decides whether cancer cells migrate or proliferate (16). Again, interaction of the EphB2 tyrosine kinase receptor with focal adhesion kinase (FAK) promotes invasiveness and inhibits proliferation in glioblastoma multiforme (19).

Here, we discuss the opposite (proliferative or migratory) functions of stromal AR and the role of the upstream AR/FlnA complex in stromal cell decision to “go or grow.” Since the AR/FlnA complex drives androgen signaling toward migration and halts cell cycle, this complex might be specifically targeted, with implications in the therapy of AR-related human diseases. The potential role of non-genomic signaling activated by androgen/AR axis in cancer-associated fibroblasts (CAFs) is also discussed.

## Androgen Signaling in Stromal Cells

AR mRNA levels are very similar in the stroma and epithelium, although AR-mediated transcription is differentially regulated in each, with a more active co-regulator recruitment by epithelial AR.

In the early 2000s, we made the exciting finding that NIH3T3 cells and mouse embryo fibroblasts (MEFs) harbor a transcriptionally incompetent AR (20). Further expansion of the study led to the discovery that AR plays a dual and opposite role in mesenchymal and transformed mesenchymal cells. At sub-optimal androgen concentration (1 pM) AR mediates cell cycle progression and proliferation, while at optimal androgen concentration (10 nM) the receptor halts mitogenesis and fosters migration (10, 11, 20). The dichotomous effect is not restricted to immortalized (NIH3T3 cells) or transformed (HT1080 cells) fibroblasts, but can also be observed in primary fibroblasts from adult or embryonic mouse (11).

A few years ago, by recombination of prostate stromal WPMY-1 cells with PCa epithelial PC-3 cells in a mouse model, Niu and colleagues showed that stromal but not epithelial AR promotes tumor proliferation at very early stage (21). Noteworthy, as occurs in fibroblasts and transformed fibroblasts, prostate stromal WPMY-1 cells harbor a transcriptionally incompetent AR, which is permanently localized in cytoplasm (22). Thus, the receptor expressed in WPMY-1 cells likely promotes growth of epithelial PC-3 cells through a non-transcriptional mechanism.

Prostate CAFs express AR, although the role of this receptor in prostate tumorigenesis is still unclear. Loss of AR expression in the tumor stroma, but not in the surrounding normal prostatic stroma tissue, increases the risk of relapse following radical prostatectomy (23, 24). Stromal AR expression progressively decreases during PCa progression (22, 23). Thus, loss of stromal AR might enable the growth, progression, and response to the therapy in PCa. In apparent contrast with these findings, tissue-specific knockout approach showed that deletion of AR in fibroblasts and fibro-muscular cells (dARKO) inhibits the growth of PCa in mouse model (6). A similar conclusion was reached using immortalized CAFs from PCa biopsies (25). By using these experimental settings, it has been shown that stromal AR modulates the release of pro-inflammatory chemokines by CAFs, thereby promoting recruitment of inflammatory and immune cells in tumor microenvironment (6, 25). These events may result in extracellular matrix remodeling and angiogenesis, as well as proliferation and invasion of neighboring epithelial PCa cells. In conclusion, conflicting findings on the role of stromal AR can be obtained using different experimental settings (i.e., tissue recombination in immune-depressed mouse or AR tissue-specific knockout in immune-intact mouse). Thus, further analysis of stromal AR should be undertaken to gain valuable preclinical findings. Moreover, the contribution of AR transcriptional and/or non-transcriptional activity to biological responses in CAFs still remains elusive.

**Figure 1:**
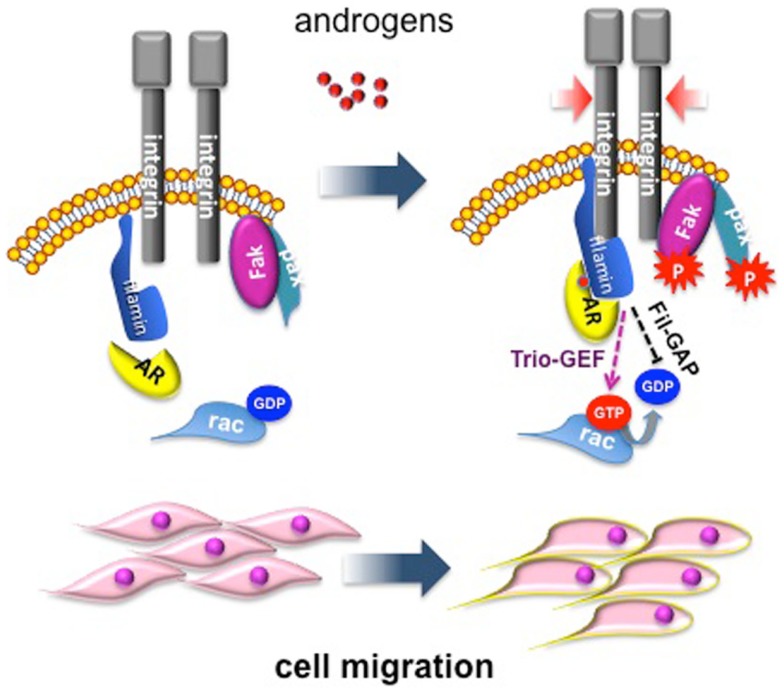
**Migratory pathway activated by androgens in mesenchymal and transformed mesenchymal cells**. Androgen (10 nM) enhances AR/FlnA co-localization at intermediate cytoskeleton filaments and induces a tripartite complex including AR/FlnA/integrin beta1 in NIH3T3 fibroblasts. This complex, likely through Trio-GEF, activates Rac. The Fln-associated GTPase activating protein (Fil-GAP) might switch off Rac-GTP. The AR/FlnA/integrin beta1 complex also triggers FAK and paxillin tyrosine phosphorylation. Activation of this machinery modifies cytoskeleton actin and cell adhesion, thus increasing motility of target cells (10).

We have dissected in recent years the pathways activated by different androgen concentrations in fibroblasts, and this approach has revealed some fascinating aspects. Low androgen concentration (1 pM) induces proliferation in NIH3T3 fibroblasts as a consequence of AR association with Src and p85, the regulatory subunit of PI3-K (20). This complex activates MAPKs and Akt, which then increase cyclin D1 expression and re-localize p27 to cytoplasm for its subsequent degradation (11, 20). Conversely, stimulation of NIH3T3 fibroblasts with optimal (nanomolar) androgen concentration does not induce AR association with Src and p85, but triggers AR association with the actin-binding protein, FlnA. Ten nM androgen stabilizes AR/FlnA co-localization at intermediate cytoskeletal filaments and induces a complex including AR/FlnA/integrin beta1 in NIH3T3 fibroblasts. This complex triggers FAK and paxillin tyrosine phosphorylation. Furthermore, the AR/FlnA/integrin beta1 complex activates Rac. These events lead to cytoskeleton reorganization, adhesion changes, and cell migration [(10); Figure 1].

On this basis, we may hypothesize that AR-mediated activation of MAPK induces the release of chemokines and growth factors by CAFs when androgen levels are low. Indeed, MAPK activation increases growth factor release in various cancer cell types [(26) and refs therein; (27)]. Again, by activating PI3-K at low androgen concentration, stromal AR may act in concert with NF-kappa-B to induce the release of macrophage inflammatory protein-1 beta (MIP-1beta) in tumor microenvironment, or it may activate IKK-alpha-dependent gene transcription. Stromal AR co-operates with NF-Kappa-B to modulate the gene expression of MIP-1beta (6), and nuclear receptors induce IKK-alpha-dependent epigenetic modifications and gene expression changes through activation of PI3-K pathway (28, 29). When androgen levels increase, CAFs might move toward epithelial PCa cells as a consequence of the AR/FlnA complex assembly and activation of molecular machinery involved in cell motility.

In conclusion, non-genomic functions of stromal AR might either sustain the proliferation and invasiveness of epithelial PCa cells or induce the recruitment of CAFs toward neighboring epithelial PCa cells. Androgen levels in tumor microenvironment may thus dictate the type of biological outcome in CAFs.

We recently showed that by increasing androgen concentration from 1 pM to 10 nM, AR shifts from Src/PI3-K toward association with FlnA, which in turns mediates the ligand-dependent activation of a different set of effectors (e.g., Trio-GEF, Rac) in stromal cells. Thus, AR-protein interactions regulate the type of hormonal responses, and these interactions are in turn controlled by ligand concentration. Different androgen levels might, for instance, affect post-translational modifications of AR. Phosphorylation, sumoylation, acetylation, and ubiquitination are potential reversible mechanisms affecting AR stability, localization, and interactions of the receptor with other proteins [reviewed in Ref. (30, 31)]. By enhancing the receptor interaction with signaling effectors, post-translational modifications might impact the internalization route of AR and receptor functions. In sum, there are different potential routes for androgens to orchestrate association of AR with Src/PI3-K or FlnA, thus providing a mechanism for downstream pathway regulation.

**Figure 2:**
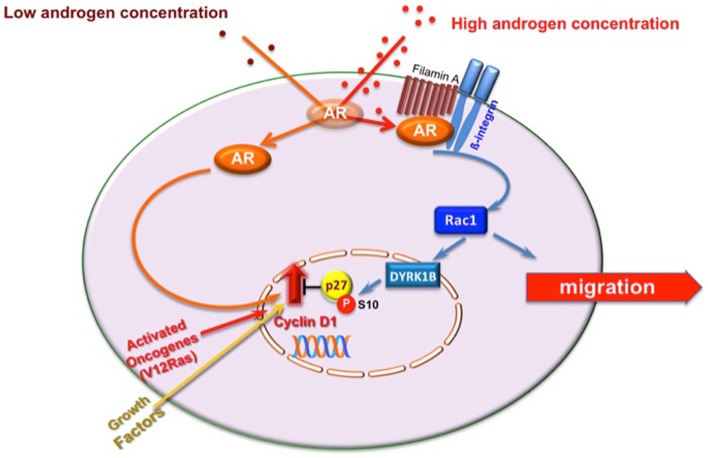
**Model of proliferative/migratory pathways activated by androgens in mesenchymal and transformed mesenchymal cells**. One pM androgen triggers cell proliferation through the AR/Src/p85alpha complex assembly (20). Ten nM androgen triggers the AR/FlnA complex assembly that in turn activates Rac. Rac enhances cell migration (10) and triggers DYRK 1B activation (11). This results in p27 Ser10 phosphorylation and p27 stabilization. Quiescence of fibroblasts and human fibrosarcoma cells then follows (11). In AR-expressing cells, oncogenic Ras or excessive growth factor stimuli increase cyclin D1 expression and cell proliferation. By stimulating DYRK 1B activation, 10 nM androgen triggers Ser10 phosphorylation and stabilization of p27 (11). This event likely counteracts the effect of activated Ras or growth factors on cyclin D1 and cell proliferation.

Migration rarely occurs in proliferating cells, and signals stimulating migration inhibit cell proliferation [reviewed in Ref. (32)]. We recently identified androgen-activated Rac as the switch regulating transition from proliferative to migratory phenotype in NIH3T3 fibroblasts and HT1080 fibrosarcoma cells. In these cells, Rac activation by 10 nM androgen halts cell cycle progression and triggers cell motility (11). Molecular studies have shown that these cells make the decision to halt cell cycle by triggering activation of the Rac-dependent DYRK 1B kinase upon challenging with 10 nM androgen. Once activated, DYRK 1B kinase triggers Ser10 phosphorylation of p27 and its stabilization. This pathway, which is controlled by the upstream AR/FlnA complex, fosters cell quiescence, and even inhibits transformation induced by oncogenic Ras [(11); Figure 2]. Thus, AR mediates growth suppression by specifically targeting Ras-driven growth-promoting pathways, highlighting the role of androgen/AR axis in human cancers. Oncogenic Ras mutations have been described in carcinomas of the pancreas, colon, lung, and thyroid, as well as in myeloid leukemia (33). Interestingly, most of these tumors (pancreas, colon, and lung) express AR (3, 34). Proliferation of various pancreatic cancer-derived cells, which express AR, is insensitive to androgens (35), and androgens inhibit survival signals in colon cancer *in vitro* and *in vivo* (36). Again, small-cell lung carcinoma H1184 cell line exhibits significant growth upon stimulation with sub-optimal androgen concentrations, but is growth-inhibited at higher androgen concentrations. Androgens also impair the growth of non-small-cell lung carcinoma H1993 cell line (37). Human fibrosarcoma HT1080 cells, exhibiting an activated N-Ras, do not grow in response to high androgen concentrations (11, 34). Hence, the arguments put forward here raise the possibility that androgens elicit anti-proliferative signals in human cancers bearing oncogenic Ras mutations through activation of DYRK 1B. Noteworthy, DYRK 1B is an active kinase in various human cancers and regulates Ras-driven transformation and tumor progression (38–40). Thus, by activating DYRK 1B kinase, androgen/AR axis might restrain Ras-driven transformation.

Stromal AR promotes the growth and differentiation of developing prostate, while it appears to inhibit the growth of PCa under certain conditions (22). We recently observed that treatment of androgen-stimulated mouse and MEFs, as well as NIH3T3 and HT1080 cells with the anti-androgen bicalutamide (Casodex) increases cell proliferation. By displacing the androgen binding to AR, bicalutamide inhibits AR-elicited migratory signals and enables AR-mediated cell proliferation through the prevention of AR/FlnA complex assembly (11). This finding is of interest, since bicalutamide is frequently used in human PCa as ADT and often promotes PCa progression [reviewed in Ref. (7)]. Therefore, our recent results in migration/proliferation of mesenchymal and transformed mesenchymal cells might partly clarify the action of bicalutamide in enhancing PCa progression through activation of undesired pathways in stromal cells. These data further suggest that novel approaches are urgently needed for PCa treatment.

In summary, our results show that upon 10 nM androgen-triggered AR/FlnA complex assembly, FlnA acts as a scaffold for Rac and effectors of its dependent pathway, thereby enabling Rac activation and recruitment by Rac of specific signaling proteins (e.g., DYRK 1B). These events lead to cell motility and simultaneous cell cycle arrest in mesenchymal and transformed mesenchymal cells. Interference in AR/FlnA complex assembly by new molecules, such as AR-derived peptides (11), may represent a promising approach to the therapy of PCa by specifically modulating signaling pathways activated by AR in stromal cells.

## Conflict of Interest Statement

The Review Editor Daniela Pasquali declares that, despite being affiliated to the same institution as the authors, the review process was handled objectively and no conflict of interest exists. The authors declare that the research was conducted in the absence of any commercial or financial relationships that could be construed as a potential conflict of interest.
